# Targeting of phagolysosomes containing conidia of the fungus *Aspergillus fumigatus* with polymeric particles

**DOI:** 10.1007/s00253-022-12287-1

**Published:** 2022-12-08

**Authors:** Katherine González, Gauri Gangapurwala, Julien Alex, Antje Vollrath, Zoltán Cseresnyés, Christine Weber, Justyna A. Czaplewska, Stephanie Hoeppener, Carl-Magnus Svensson, Thomas Orasch, Thorsten Heinekamp, Carlos Guerrero-Sánchez, Marc Thilo Figge, Ulrich S. Schubert, Axel A. Brakhage

**Affiliations:** 1 Department of Molecular and Applied Microbiology, Leibniz Institute for Natural Product Research and Infection Biology – Hans Knöll Institute (Leibniz-HKI), Adolf-Reichwein-Straße 23, 07745 Jena, Germany; 2 Institute of Microbiology, Friedrich Schiller University Jena, Neugasse 25, 07745 Jena, Germany; 3 Laboratory of Organic and Macromolecular Chemistry (IOMC), Friedrich Schiller University Jena, Humboldtstraße 10, 07743 Jena, Germany; 4 Jena Center for Soft Matter (JCSM), Friedrich Schiller University Jena, Philosophenweg 7, 07743 Jena, Germany; 5 Applied Systems Biology, Leibniz Institute for Natural Product Research and Infection Biology – Hans Knöll Institute (Leibniz-HKI), Adolf-Reichwein-Straße 23, 07745 Jena, Germany

**Keywords:** *Aspergillus fumigatus*, PLGA particle, Phagolysosome fusion, Particle delivery, Vacuolin1

## Abstract

**Abstract:**

Conidia of the airborne human-pathogenic fungus *Aspergillus fumigatus* are inhaled by humans. In the lung, they are phagocytosed by alveolar macrophages and intracellularly processed. In macrophages, however, conidia can interfere with the maturation of phagolysosomes to avoid their elimination. To investigate whether polymeric particles (PPs) can reach this intracellular pathogen in macrophages, we formulated dye-labeled PPs with a size allowing for their phagocytosis. PPs were efficiently taken up by RAW 264.7 macrophages and were found in phagolysosomes. When macrophages were infected with conidia prior to the addition of PPs, we found that they co-localized in the same phagolysosomes. Mechanistically, the fusion of phagolysosomes containing PPs with phagolysosomes containing conidia was observed. Increasing concentrations of PPs increased fusion events, resulting in 14% of phagolysosomes containing both conidia and PPs. We demonstrate that PPs can reach conidia-containing phagolysosomes, making these particles a promising carrier system for antimicrobial drugs to target intracellular pathogens.

**Key points:**

• *Polymer particles of a size larger than 500 nm are internalized by macrophages and localized in phagolysosomes*.

• *These particles can be delivered to Aspergillus fumigatus conidia-containing phagolysosomes of macrophages*.

• *Enhanced phagolysosome fusion by the use of vacuolin1 can increase particle delivery*.

**Supplementary Information:**

The online version contains supplementary material available at 10.1007/s00253-022-12287-1.

## Introduction

Macrophages phagocytose invading microorganisms, which finally end up in a hostile organelle, i.e., the phagolysosome. This organelle is characterized by an acidic pH value, the presence of lytic enzymes, and a high concentration of reactive oxygen and nitrogen intermediates (Pauwels et al. 2017). However, several microorganisms, including the tuberculosis-causing bacterium *Mycobacterium tuberculosis* or conidia of the important human-pathogenic fungus *Aspergillus fumigatus*, survive their phagolysosomal localization by interfering with the function and/or maturation of phago(lyso)somes (Schmidt et al. 2020; Queval et al. 2017; Heinekamp et al. 2015). In this intracellular niche, these pathogens can hide from the residual immune system and are also difficult to reach with the existing antimicrobials that have to cross two membranes*,* i.e., the cytoplasmic and the phagosomal membrane (Heinekamp et al. 2015; Queval et al. 2017; Kamaruzzaman et al. 2017)*.* Therefore, for the efficient killing of such intracellular microorganisms, antimicrobials must reach phagosomes or even phagolysosomes. Polymeric particles (PPs) are promising carrier systems for the delivery of antimicrobial drugs to immune cells (Donnellan and Giardiello 2019; Kumar et al. 2017; Lee et al. 2015; Mejía et al. 2021) and potentially to phagosomes. However, before such drug delivery systems can be further developed, it is necessary to assess whether a PP can reach a phagolysosome containing a pathogenic microorganism.

Here, we address this fundamental question by analyzing the conidia of *A. fumigatus* residing in phagolysosomes. *A. fumigatus* is a ubiquitous filamentous fungus, which can cause severe infections in humans, such as invasive aspergillosis, aspergilloma, or disseminated infections, particularly in immunocompromised patients (Brown et al. 2012; Brakhage 2005). The incidence of life-threatening invasive aspergillosis has been estimated to be more than 300,000 cases per year worldwide with a mortality rate of up to 90% depending on the patient cohort (Bongomin et al. 2017; Brakhage 2005; Lin et al. 2001). The most important risk factors for the development of invasive infections are immune deficiencies, e.g., due to hematological malignancies, solid organ transplantation, or immunosuppressive corticosteroid therapy (Latgé and Chamilos 2019).

*A. fumigatus* produces small conidia with a size range of 2 to 3 µm which are released into the air and thereby distributed in the environment (Deacon et al. 2009). After inhalation, resident alveolar macrophages in the lung phagocytose and degrade foreign particulate matter or microorganisms, e.g., fungal conidia, in phagolysosomes (Heinekamp et al. 2015; Latgé and Chamilos 2019). However, *A. fumigatus* has established mechanisms to survive in these organelles (Heinekamp et al. 2015). The conidial pigment consisting of dihydroxynaphthalene (DHN) melanin interferes with the phagosomal maturation (Thywissen et al. 2011; Jahn et al. 2002) by inhibiting the formation of lipid rafts, which are required for the assembly of ATPase and NADPH oxidase (NOX) complexes on the phagolysosomal membrane (Schmidt et al. 2020, 2018). These and other mechanisms allow conidia to extend their persistence within phagosomes and eventually germinate to escape from macrophages and invade host tissue.

Current therapies to treat invasive aspergillosis include the use of echinocandins, triazoles, and polyenes (Valiante et al. 2015; Panackal et al. 2014). However, treatment with these available drugs is hampered due to complications associated with therapeutic approaches, including pharmacological interactions with co-administered drugs, toxicity, and intolerance (Valiante et al. 2015; Panackal et al. 2014; Denning and Hope 2010; Kischkel et al. 2020; Italia et al. 2011). Therefore, the treatment of systemic infections with encapsulated antifungals has been widely explored in the last decades, and different formulations have successfully demonstrated superiority *versus* pristine drugs (Kischkel et al. 2020). A well-known example is Ambisome®, a liposomal formulation for the delivery of amphotericin B, which has been further improved by the use of polymers (Italia et al. 2011). Polymer-based nanoparticles loaded with amphotericin B have shown promising results for peroral delivery and antileishmanial activity (Italia et al. 2011; Palma et al. 2018). In addition, other antifungals such as itraconazole have shown improved delivery and antifungal activity when encapsulated in PPs (Bian et al. 2013; Mejía et al. 2021). Therefore, targeting intracellularly persisting conidia using PPs would open up a novel avenue to treat infections with potentially reduced side effects.

Particles with a size above 500 nm are known to be phagocytosed by immune cells including macrophages (Champion et al. 2008). The internalization of PPs can be well monitored by microscopy using fluorescence-labeled biodegradable particles (Vollrath et al. 2013; Ruedas-Rama et al. 2012). These biodegradable PPs often consist of poly(lactic-*co*-glycolic acid) (PLGA), a copolymer approved by the Food and Drug Administration (FDA) for commercial formulations and acknowledged to be potentially useful to deliver antimicrobials against intracellular infections (Zhong et al. 2018; Danhier et al. 2012). PLGA is degraded into non-toxic lactic and glycolic acids, which either enter the tricarboxylic acid cycle or can be excreted via the kidneys (Zweers et al. 2004). Various techniques can be utilized for the formulation of PLGA particles depending on the desired size and intended application (Palma et al. 2018; Patel et al. 2011; Mejía et al. 2021). Nanoprecipitation, single or double emulsion, and the salting-out method are some of the commonly adopted techniques (Sah and Sah 2015; Nava-Arzaluz et al. 2012; Rosca et al. 2004).

Here, we report the formulation of fluorescent-labeled PLGA microparticles with a size of approximately 800 nm to enable their phagocytosis and intracellular processing in macrophages. Furthermore, we demonstrate as a proof-of-concept that PLGA PPs can reach microbe-containing phagolysosomes making these particles a promising carrier system for antimicrobial drugs to target intracellular persistent pathogens.

## Methods

### Formulation and characterization of particles

Synthesis of PLGA-DY-550 is available in the Supplementary Information. PLGA-DY-550 particles were formulated by single emulsion technique using a T25 digital ULTRA-TURRAX® (IKA, Staufen, Germany), a high-speed homogenizer equipped with a 10 mm diameter tip. PLGA-DY-550 was dissolved in ethyl acetate with a concentration of 50 mg mL^−1^ to form an oil phase (O) and 0.5 mL of the solution were mixed with 2.5 mL of an aqueous phase (W1) containing 1.5% (w/v) of poly(vinyl alcohol) PVA. To this biphasic solution, high-speed homogenization was applied to both phases (W1/O) at a speed of 12,000 rpm for 60 s. After droplet formation, 2 mL of Milli-Q water were added to stabilize the particle suspension and to reach a particle concentration of 5 mg mL^−1^. The suspension was left to evaporate for at least 4 h to eliminate the organic solvent. To further remove the excess amount of PVA, the particles were centrifuged at 3354 g for 15 min and resuspended in 4 mL of Milli-Q water. Subsequently, 1 mL of this suspension was lyophilized to determine the concentration by weighing the lyophilized powder (Supplementary Table S1) (Morais et al. 2016). The PVA content in the final formulation was determined using the protocol briefly described in Supplementary Table S1 and Fig. S1. These samples were then subjected to absorbance measurements at λ = 650 nm using the TECAN Infinite M200pro microplate reader (Tecan, Männedorf, Switzerland).

The characterization of final formulations in terms of size (Z-average), size distribution, and polydispersity index (PDI) along with zeta potential (ζ) was determined using a Zetasizer Nano ZS device (Instruments, Malvern, UK) based on dynamic light scattering (DLS) that measures photons backscattered at 173° (Perevyazko et al. 2010) with a laser wavelength of λ = 633 nm, and electrophoretic light scattering (ELS) techniques. Briefly, 10 µL of the sample was diluted with 100 µL of pure water and measured at 25 °C after 30 s of equilibration time (*n* ≥ 3) for 30 s. To calculate the Z-average (hydrodynamic diameter d_H_) and the PDI values, the cumulant method (Malvern 2013) was used based on the refractive index (1.330) and viscosity (0.8872 cP) of water. Similarly, for obtaining zeta potential values (ζ), 10 µL of the initial particle suspension (*c* = 5 mg mL^−1^) was diluted in 1 mL of pure water and measured at 25 °C in a DTS1060 cell (Malvern Instruments) with the automatic mode after 30 s of equilibration time.

For scanning electron microscopy (SEM) imaging of particles, 10 µL of the PPs suspension was dropped on a mica surface and dried for 4 h at room temperature (RT). The dried samples were coated with a thin layer of platinum with a CCU-010 HV (Safematic, Zizers, Switzerland) and measured under a Sigma VP field emission SEM (Zeiss, Jena, Germany) using the InLens detector with an accelerating voltage of 6 to 8 kV.

### Internalization of particles by macrophages

RAW 264.7 macrophages (ATCC TIB-71) were incubated overnight in a 6-well plate with a cell density of 3 × 10^6^ cells per well (conditions described in the Supplementary Information for the evaluation of cytotoxicity). PPs were added into each well every hour for 6 h at a concentration of 10 µg mL^−1^ in 3 mL Dulbecco’s modified Eagle medium (DMEM) containing 2% (v/v) of fetal calf serum (FCS). The cells were centrifuged at 100 g for 5 min after every PPs addition. After incubation for 6 h, the cells were washed with phosphate-buffered saline (PBS) and detached using 0.5 mL of TrypLE (Thermo Fisher, Dreiech, Germany) per well for 5 min. Finally, for fixation, the cell suspensions were centrifuged for 2 min at 600 g at 4 °C and subsequently resuspended in 4% (v/v) formaldehyde prepared in PBS for 15 min at RT and washed twice. Imaging flow cytometry, using the Amnis ImageStreamX Mk II (Luminex, Austin, TX, USA), measured 3000 to 15,000 cells and the spot count-guided analysis from the software IDEAS (https://www.softwareideas.net/) was used for data analysis. The gating strategy is described in detail in Supplementary Fig. S2. Additionally, to determine the uptake of higher concentrations of particles, PP suspensions were tested in concentrations of 50 and 100 µg mL^−1^ following the above-mentioned procedure and it was incubated for 2 h.

### Immunofluorescence staining of phagolysosomes containing PPs

RAW 264.7 macrophages were incubated in 8-well Millicell slide chambers (Merck Millipore, Burlington, MA, USA) overnight with a density of 1 × 10^5^ cells per well. For lysosomal-associated membrane protein 1 (Lamp1) immunofluorescent staining, *A. fumigatus* CEA17ΔakuB conidia (Da Silva Ferreira et al. 2006; Bertuzzi et al. 2021; the strain can be obtained from the Fungal Genetics Stock Center under strain number FGSC A1151; its parental strain is CEA10 and can be obtained from the Centraalbureau voor Schimmelcultures under the number CBS 144.89) were harvested and stained with fluorescein isothiocyanate (FITC), as described elsewhere (Kraibooj et al. 2015; Thywissen et al. 2011). Macrophages were infected with FITC-labeled conidia at a multiplicity of infection (MOI) of 5 and were centrifuged for 5 min at 100 g to synchronize their uptake. Then, the cells were incubated for 2 h at 37 °C and 5% (v/v) CO_2_ under humidified atmosphere. Before adding particles to the conidia-containing macrophages, non-phagocytosed conidia were stained with calcofluor white (CFW) (final concentration of 100 µg mL^−1^) for 2 min at RT, followed by rinsing the cells twice with PBS (Thywissen et al. 2011; Cseresnyes et al. 2020); 10 µg mL^−1^ of PPs was added to the cells in a fresh medium with 2% (v/v) of FCS. Then, potential fusion enhancers were added to the following final concentrations: Metformin 2 mM, IFN-γ 200 U mL^−1^, chloroquine 10 µg mL^−1^ (tested together with 10 µg mL^−1^ PPs), and vacuolin1 1 µM, apilimod 100 nM, wortmannin 10 nM, and dimethyl sulfoxide (DMSO) (tested together with 100 µg mL^−1^ PPs). The samples were centrifuged for 5 min at 100 g to synchronize the uptake and incubated for 4 h at 37 °C and 5% (v/v) CO_2_ under humidified atmosphere. Thereafter, the cells were washed and fixed with 4% (v/v) formaldehyde in PBS for 15 min at 20 °C. Before Lamp1 immunofluorescent staining, the cells were incubated for 30 min at 20 °C in a blocking and permeabilizing solution containing 1% (w/v) of bovine serum albumin (BSA), 0.5% (w/v) of saponin, and 22.52 mg mL^−1^ of glycine in 0.1% (v/v) Tween 20 in PBS. For Rab7 immunofluorescent staining, the cells were infected as described above for Lamp1. After incubation with particles, the cells were washed and fixed with 4% (v/v) formaldehyde in PBS for 15 min at 20 °C. The blocking solution to reduce the unspecific binding of the Rab7 antibody consisted of PBS (pH 8.0), 0.1% (v/v) of Triton™ X-100 and 1% (w/v) of BSA. Primary antibodies (diluted 1:100) from rabbit anti-mouse Lamp1 and rabbit anti-mouse Rab7, respectively, were added to the blocking solution and were separately incubated with the cells overnight at 4 °C. Incubation with the secondary antibody goat anti-rabbit DyLight 633 (Thermo Fisher) was executed in the blocking solution for 1 h at 37 °C. After antibody addition, the cells were washed thrice with PBS. The samples were then covered with a mounting medium and coverslips for microscopy. A scheme of the strategy used can be found in the supplementary information (Supplementary Fig. S3). Confocal laser scanning microscopy (CLSM) with a Zeiss LSM 780 (Zeiss) with a Plan-Apochromat 63 × /1.40 oil DIC M27 objective was used to collect images at 73 nm per pixel resolution, 1024 × 1024 pixels per image at 16-bit depth. Details on the method used to identify the particles in conidia-containing phagolysosomes by transmission electron microscopy (TEM) can be found in the Supporting Information.

### Quantification of PPs in conidia-containing phagolysosomes 

RAW 264.7 macrophages were incubated in Millicell slides and infected as described for immunofluorescence staining. The PP suspension was added in concentrations of 10 µg mL^−1^, 50 µg mL^−1^, and 100 µg mL^−1^ in DMEM with 2% (v/v) FCS. The cells were incubated for 4 h at 37 °C and 5% (v/v) CO_2_ under humidified atmosphere. Lamp1 was detected by immunofluorescent staining, as described above. The number of phagolysosomes containing conidia or both PPs and conidia was quantified with the ImageJ software using 10 images per sample, which corresponds to 559 (± 117) phagolysosomes per sample. Each particle concentration was analyzed seven times.

### Statistical analysis

Fitting of PP distributions and calculation of ANOVA for the dynamics of PP uptake were done in R version 4.0.1 (Team RC 2020). The negative binomial (NB) distributions were fitted using the fitdistrplus library (Delignette-Muller and Dutang 2015), and the logistic curves were fitted with the aid of a non-linear least squares algorithm. Analysis scripts are available from the authors upon request. The correlation between the percentage of phagolysosomes containing conidia and PPs and the concentration of PPs were analyzed with the software GraphPad Prism version 5.00 for Windows (GraphPad Software, www.graphpad.com), using Pearson correlation two-tailed (95% of confidence). The data obtained for cytokine secretion, percentage of phagolysosomes containing both conidia and particles of different sizes, and percentage of phagolysosomes containing both conidia and particles treated with inhibitors were analyzed by one-way ANOVA with multiple comparisons, using the same GraphPad software aforementioned.

## Results

### Generation of fluorescent-labeled PPs with diameters > 500 nm

**Fig. 1:**
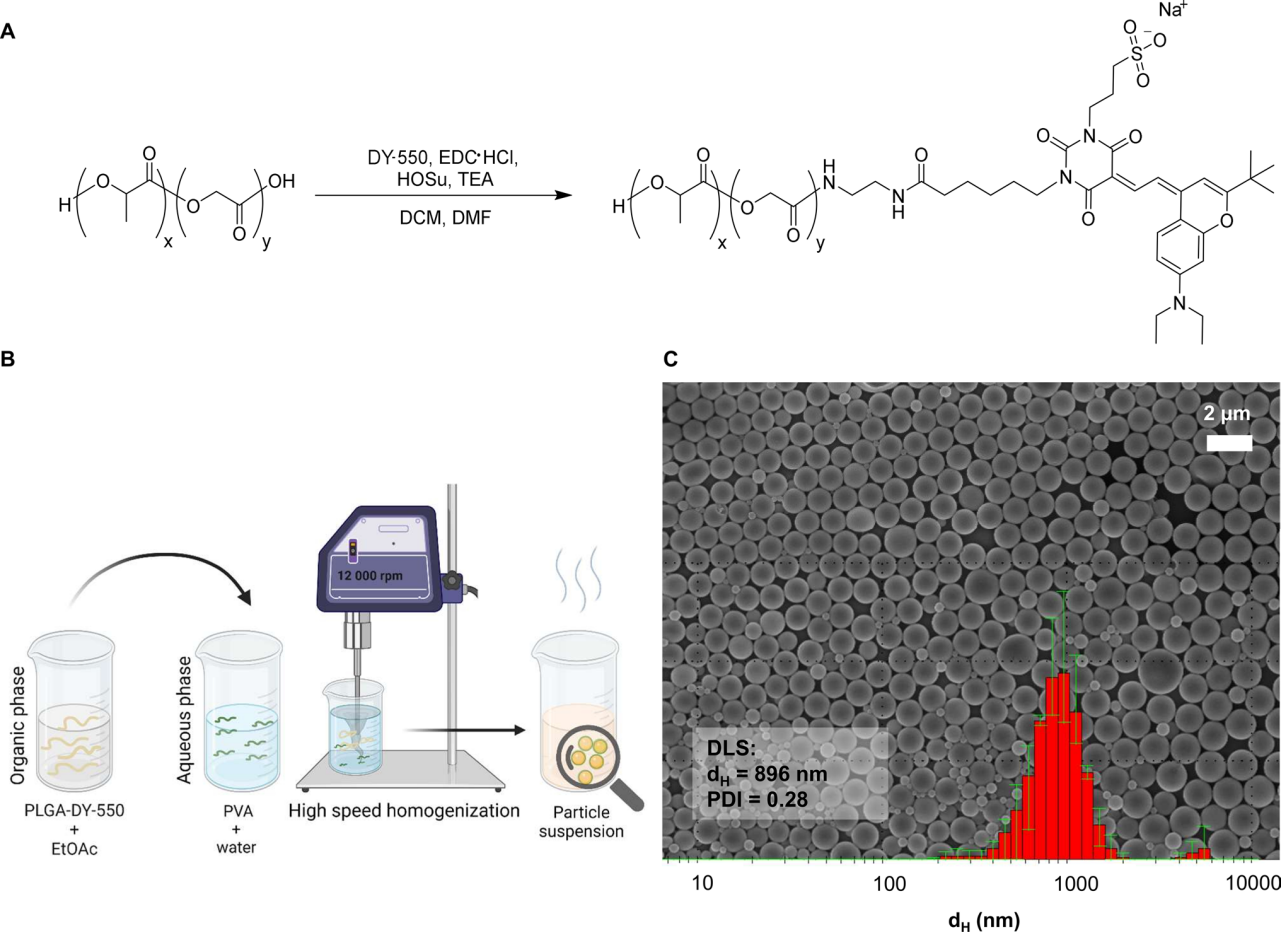
Polymer synthesis and particle formulation. (**A**) Schematic representation of the labeling of PLGA with the amino-functionalized DY-550 by 1-ethyl-3-(3-dimethylaminopropyl)carbodiimide (EDC) coupling. (**B**) Schematic representation of PLGA-DY-550 particle formulation utilizing high-speed homogenization. (**C**) SEM image of particles from batch PP 2 (batch information in Supplementary Table S1), intensity size distribution as well as d_H_ and PDI values obtained in suspension by DLS with the cumulant analysis method

To prevent uncontrolled leakage of a dye being only encapsulated in particles (Snipstad et al. 2017), here, an amino derivative of dye DY-550 was covalently attached to acid-terminated PLGA, as schematically depicted in Fig. 1A. Size-exclusion chromatography measurements with a diode array detector (DAD) confirmed the covalent attachment as well as the absence of uncoupled dye in the purified polymer (Supplementary Fig. S4A–C). Additional spectroscopic characterization of DY-550 functionalized PLGA showing the integrity of the fluorophore after the covalent coupling can be found in Supplementary Fig. S4D,E.

For the formulation of large PLGA particles, high-speed homogenization via ULTRA-TURRAX® was applied. Ethyl acetate was selected as a highly volatile and class 2 solvent and PVA as a water-soluble and non-toxic polymer surfactant with an initial concentration of 1.5% (w/v) (Fig. 1B) (Ben Halima 2016; Solaro et al. 2000; Mejía et al. 2021). After the formation of the emulsion, PPs were washed and collected by centrifugation with a subsequent re-suspension in water to remove excess PVA. The final suspensions were characterized by DLS, ELS, and SEM to determine the size distribution, surface charge, and shape of the PPs. The sizes of PPs determined by DLS ranged between d_H_ = 700 nm and 900 nm with PDI values of about 0.3 (Supplementary Table S1). This is supported by SEM imaging revealing spherical particles in the expected size range (Fig. 1C and Supplementary Fig. S5). The ζ of the PPs was found to be in the range of − 20 to − 40 mV, which indicates a stable suspension due to the highly negative value that leads to electrostatic repulsion between the particles (Singh and Lillard 2009).

### PPs are internalized by macrophages and localized in phagolysosomes

**Fig. 2:**
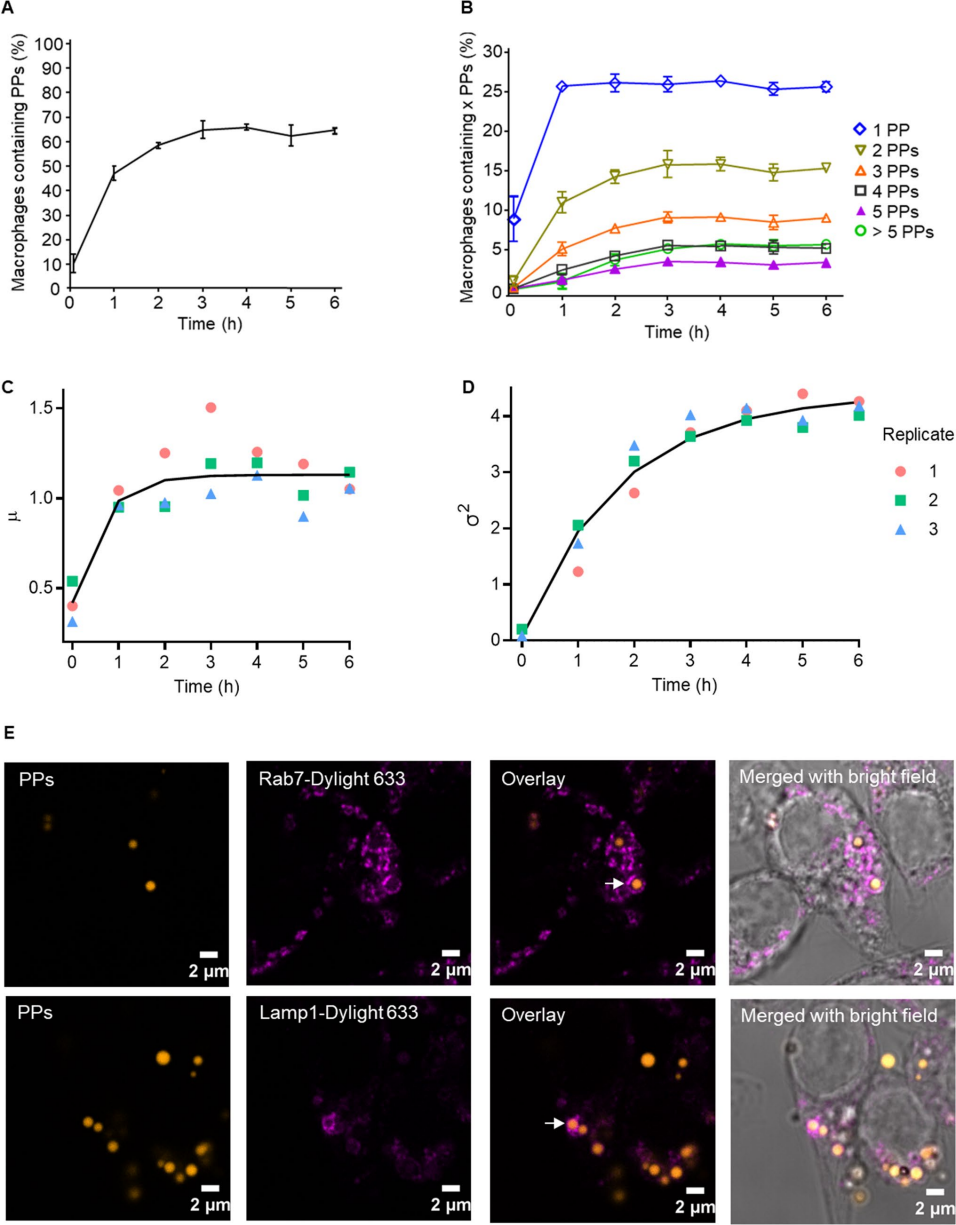
Uptake kinetics of PPs by macrophages. (**A**) Percentage of macrophages with internalized PPs vs. time. Time point 0 h represents 5 min after centrifugation. 10 μg mL^−1^ PPs (equivalent to ca. 10 PPs per cell) were used. Error bars represent standard deviation (*n* = 3). (**B**) Percentage of macrophages containing different numbers of PPs (as indicated) vs. time. (**C**) Development of the mean and variance (**D**) of the fitted NB distribution over a period of 6 h. Points are parameter fits of the NB distributions to each of the replicates at different time points. The line describes a logistic growth curve that describes the evolution of the parameters. (**E**) CLSM images of macrophages containing PPs (orange) in phagolysosomes positive for Rab7 and Lamp1. Phagolysosomes were stained with rabbit anti-mouse Rab7 and anti-mouse Lamp1 antibody detected with a DyLight 633-conjugated goat anti-rabbit antibody (purple). Images obtained after 4 h incubation with PPs. Co-localization of PP in Rab7- and Lamp1-positive compartments is marked by a white arrow

To investigate the potential of the prepared PPs as a drug delivery system for targeting pathogen-containing macrophages, the number of internalized particles per cell and their uptake kinetics were assessed. For this purpose, the percentage of macrophages containing PPs and the number of PPs per macrophage were measured over an incubation period of 6 h using imaging flow cytometry. To synchronize the uptake of PPs, an initial short centrifugation step of 5 min was performed directly after the addition of PPs to the cells. At this time point, considered as 0 h, already 10% of the macrophages contained at least one PP (Fig. 2A,B), reflecting an uptake during the 5 min centrifugation step after adding the PPs. After 1 h of incubation, 47% of the macrophages had internalized PPs. This further increased with time, reaching the highest uptake of 66% after 4 h (Fig. 2A). While the percentage of macrophages with one PP internalized did not change after 1 h, the percentage of macrophages with more than one PP internalized slightly increased over time (Fig. 2B).

To describe the dynamics of PPs uptake, a negative binomial (NB) distribution (Furman 2007; Bliss and Fisher 1953) was fitted to the number of PPs per macrophage for each time point. The NB distribution resembles a better fit to the data compared to a Poisson distribution based on visual inspection (Supplementary Fig. S6A), as well as a comparison using the Bayesian information criterion (Supplementary Fig. S6B) for the two distributions. To ensure that the NB distribution is not only more suitable than the Poisson distribution but also a reliable fit to the data, we evaluated quantile–quantile (Q-Q) plots for both distributions. The sample values strongly deviated from the theoretical values when a fitted Poisson model was used to produce the Q-Q plot (Supplementary Fig. S6C). In contrast, when a fitted NB distribution was used for the theoretical values, we observed a good agreement between the model and the sample (Supplementary Fig. S6D). After fitting the NB distribution to each time point, the means ($\mu$) of the variances (${\sigma }^{2}$) were calculated during the experiment. These values were plotted and are displayed in Fig. 2C, reflecting the dynamics of the uptake process. An average of ~ 0.4 PPs per macrophage was found at time point 0 h. Thereafter, the number of PPs quickly increased to a steady state level of ~ 1.1 PPs per macrophage after 2 h, and no further increase was observed. The variance of the distribution increased for at least 3 h.

To statistically quantify the dynamics, we fitted a logistic growth function to the fitted parameters of the form ${f}_{L}\left(t\right)=A\left(1-{e}^{-bt}\right)+c,$ where the parameters $\mathrm{A},\mathrm{ b}$, and $\mathrm{c}$ were simultaneously fitted for all three replicates. The fitted logistic function is plotted as a black line in Fig. 2C. Uptake saturation was tested by comparing the fitted ${f}_{L}(t)$ against the null hypothesis, i.e., that the dynamics of the parameter is constant over time. The *p*-values to test this hypothesis were obtained from the two-sided ANOVA test and are summarized in Supplementary Table S2. Based on the data summarized in Fig. 2C, the average number of PPs in the macrophages was reached within the first hour of the experiment, followed by a saturation of the system. Even though the average number of PPs per macrophage did not significantly change after 2 h (Fig. 2C), the variance of the distribution kept increasing, as observed in Fig. 2D. This finding suggests a simultaneous increase of PP numbers in some macrophages and a decrease in others, i.e., a higher variance of the distribution without a significant change in the mean reflects the equilibrium between uptake and degradation. Additionally, when higher concentrations of PPs were provided, we observed an increase in the percentage of macrophages containing particles, as well as an increase in the number of particles per cell. When using 100 µg mL^−1^ of PPs, the percentage of macrophages containing particles increased to 83.5% and the number of particles per macrophage increased to 4 after 2 h of incubation (Supplementary Fig. S7).

In addition to uptake kinetics, the intracellular localization of PPs was followed. As shown by immunofluorescence, PPs were found in phagolysosomes as indicated by a positive signal for the late endosomal marker Rab7 and the lysosomal marker Lamp1 (Fig. 2E).

### PPs are delivered to conidia-containing phagolysosomes

**Fig. 3:**
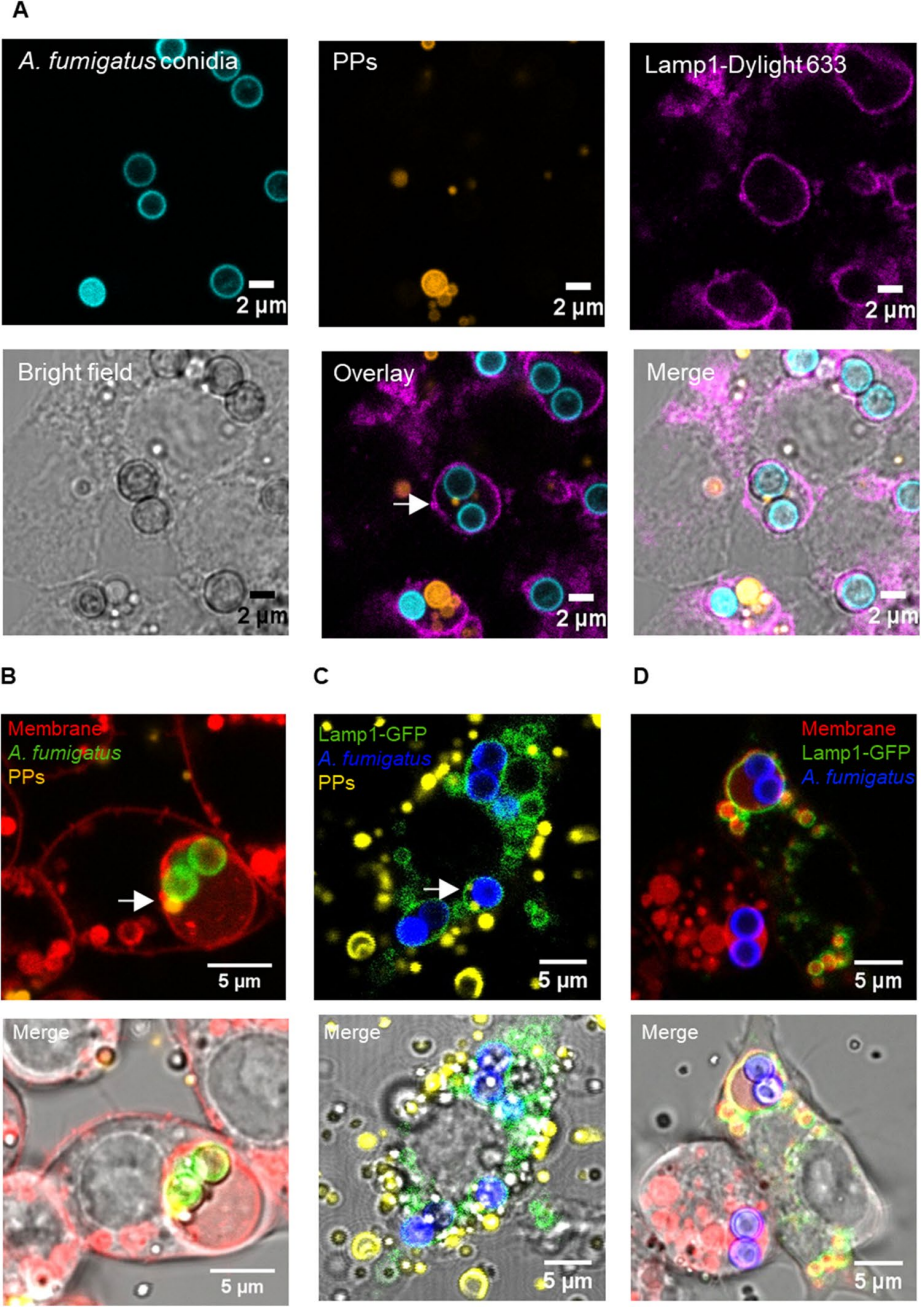
Co-localization of PPs with conidia in phagolysosomes. (**A**) PPs (orange) and conidia (cyan) were found in phagolysosomes (purple) stained by immunofluorescence with rabbit anti-mouse Lamp1 antibody and detected with goat anti-rabbit DyLight 633-conjugated antibody. All conidia were stained with FITC (cyan) prior to infection. Co-localization of a PP with two conidia is marked by a white arrow. The diameter of the corresponding PP is ~ 900 nm, which agrees well with the maximum of the PP size distribution (Fig. 1C). Detection of PPs present in phagolysosomes containing conidia by live cell imaging and co-localization of phagolysosomal membrane stained with (**B**) Cellmask™ Deep Red and (**C**) CellLight Lamp1-GFP on the phagolysosomal membrane. The images in the upper line show fluorescence images, and the images below show fluorescence plus brightfield. Infected macrophages with *A. fumigatus* conidia stained with CFW (blue) after 4 h of incubation with PPs. (**D)** Lamp1-GFP and endosomal membrane signals overlap

To investigate whether PPs can reach conidia-containing phagolysosomes, macrophages were incubated for 2 h with *A. fumigatus* conidia before the addition of PPs. Interestingly, co-localization of PPs in the conidia-containing phagolysosome was observed (Fig. 3A). Conidia not taken up by macrophages and still present in the medium before the addition of PPs were counterstained with CFW. Because CFW is not permeable through cell membranes, this step enables discrimination of extracellular conidia from already internalized conidia at the time of PP addition (Cseresnyes et al. 2020; Thywissen et al. 2011). It also allows discriminating between PPs reaching a phagolysosome that already contains a conidium and phagolysosomes that contain conidia and PPs that were simultaneously taken up and ended up in the very same phagolysosome. This way, we excluded the last mentioned in further investigations.

To further substantiate our findings, the co-localization of conidia and PPs was also analyzed by additional techniques including live cell imaging (method in the Supplementary information). By applying an unspecific membrane staining (CellMask Deep Red, Thermo Fisher) before the addition of conidia and PPs to cells, we observed co-localization of both conidia and PPs in a membrane-surrounded compartment (Fig. 3B). Furthermore, phagolysosomes were also labeled with Lamp1-GFP. For this purpose, macrophages were transiently transfected with Lamp1-GFP-encoding DNA (Fig. 3C). The CellMask Deep Red staining co-localized with the Lamp1-GFP (Fig. 3D), which strongly suggested co-localization of PPs and conidia in the very same phagolysosome.

**Fig. 4:**
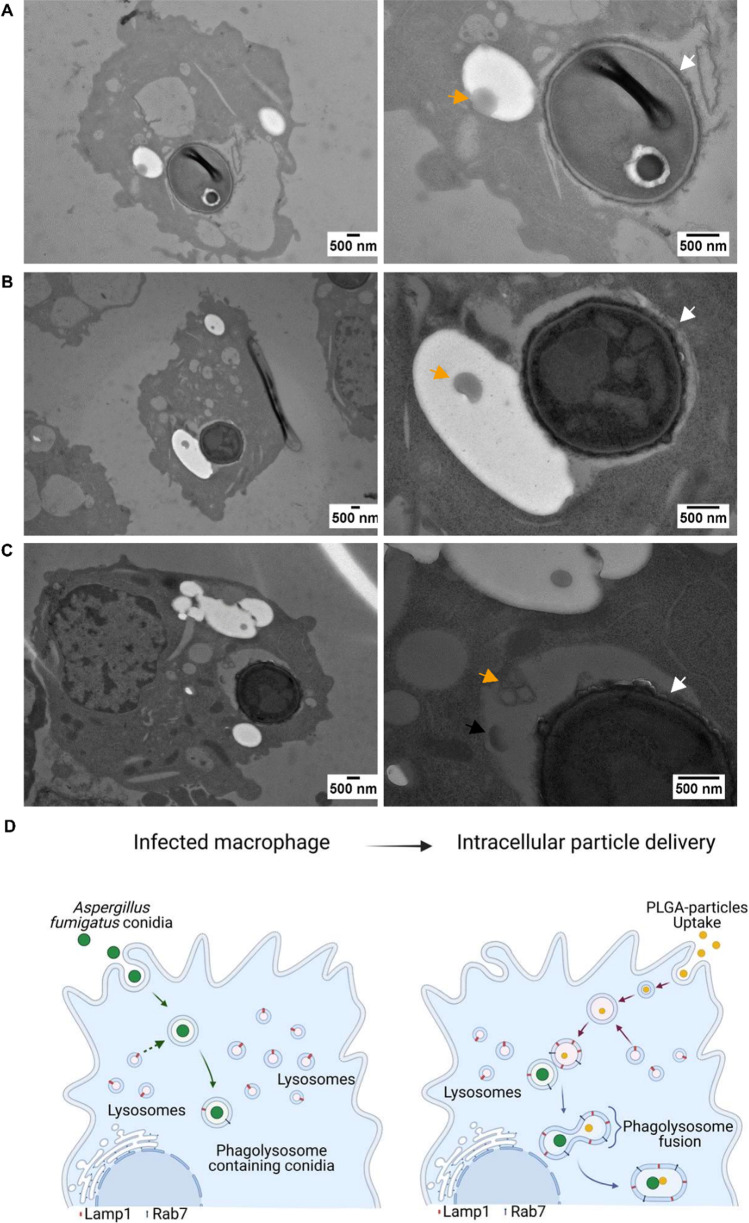
TEM images to monitor the fusion of conidia-containing phagolysosomes with PP-containing phagolysosomes. Left: overview images showing macrophages with phagocytosed conidia and PPs. Right: detail of left image showing PPs and conidia-containing phagolysosomes. (**A**) PP and conidium inside of a phagolysosome; both compartments are in close proximity. (**B**) PP in a phagolysosome containing a conidium, intraluminal contents are not yet mixed. (**C**) PPs in a conidium-containing phagolysosome. Conidia are marked by white arrows and PPs by orange arrows. The black arrow points to an apparent partially disintegrated PP. (**D**) Diagram of particle delivery to infected macrophages

To further confirm these findings, we investigated co-localization by TEM. As shown in Fig. 4A–C, the fusion of a compartment containing PPs with phagolysosomes containing conidia progressed when both membranes came in contact. Based on the applied staining conditions, compartments containing PPs are shown with bright contrast. This effect was observed due to the longer staining procedure required to improve contrast for the PLGA-based PPs, which generally only allow for weak staining. Based on the results shown in Figs. 2E and 4A, PPs were located within a single compartment surrounded by a membrane that confines a phagolysosome. The membrane of the compartment containing a particle is visible around the bright compartment. We also observed differences in the image contrast of the phagolysosomal lumen between PP-containing phagolysosomes and conidia-containing phagolysosomes (Fig. 4B). Figure 4B suggests that fusion of phagolysosomal membranes occurs, which is further supported by the image displayed in Supplementary Fig. S8, where the fusion of membranes is documented. The outcome of such a fusion event is visible in Fig. 4C, i.e., PPs co-localized with a conidium in a phagolysosome. Collectively, these data provide strong evidence that co-localization of conidia and PPs can occur, and therefore, targeting of pathogen-containing phagolysosomes with PPs is feasible, as it is depicted in Fig. 4D.

### Enhanced phagolysosome fusion increases particle delivery 

**Fig. 5:**
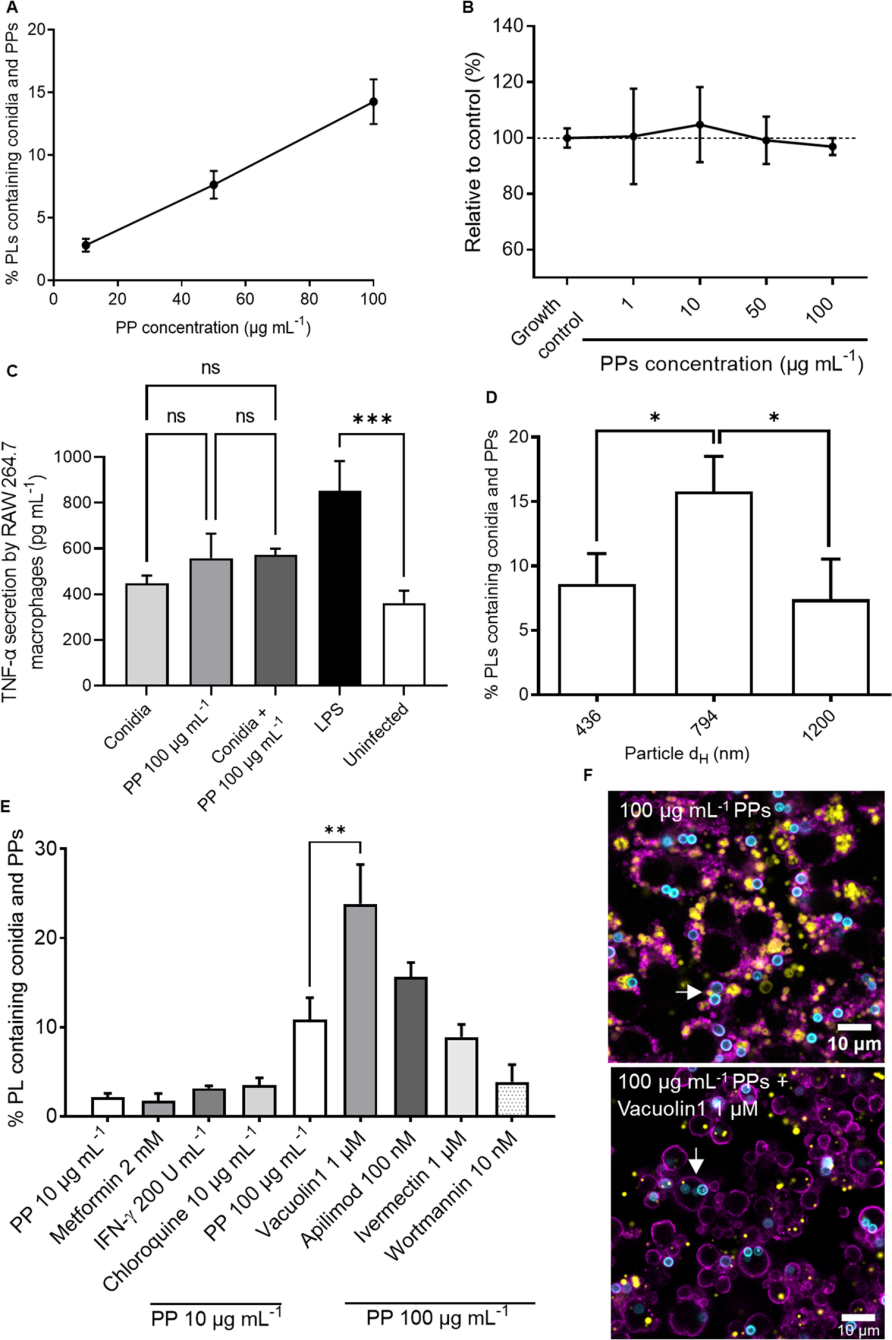
Influence of particle concentration, size and inhibitors on phagolysosomes containing both PPs and conidia after 4 h of incubation. (**A**) Percentage of phagolysosomes containing both conidia and particles, according to particle concentration. Black lines connect the dots that correspond to the mean values. Error bars represent the standard error of the mean of seven replicates. A detailed explanation of the quantification method can be found in Supplementary Fig. S3. (**B**) Cytotoxicity assay. Viability of RAW 264.7 macrophages treated with increasing concentrations of PPs determined by the metabolic activity of cells. Dashed line: 100% metabolic activity which corresponds to the untreated control cells. Error bars represent the standard deviation of the mean of three replicates. Replicates were analyzed by Student’s *t*-test. Statistical significance: *$p$ ≤ 0.05. (**C**) Tumor necrosis factor-α (TNF-α) secretion by RAW 264.7 macrophages detected by ELISA after 8 h of infection and particle addition (method described in the Supplementary Information). Error bars represent the standard deviation of the mean of three replicates. Replicates were analyzed by one-way ANOVA. Statistical significance: ***$p$ ≤ 0.001, ns: no significant difference. (**D**) Percentage of phagolysosomes containing both conidia and particles of different sizes after 4 h of incubation. Infected RAW 264.7 macrophages were treated with a concentration of 100 µg mL^−1^ PPs of each particle batch (PP 5, 6, and 7 in Supplementary Table S1). (**E**) Inhibitors of different metabolic pathways of macrophages to increase or decrease the percentage of phagolysosomes containing both particles and conidia. Error bars represent the standard deviation of the mean of three replicates. Replicates were analyzed by one-way ANOVA. Statistical significance: **$p$ ≤ 0.005. (**F**) Representative confocal microscopy image of 100 µg mL^−1^ PPs (yellow) with conidia (cyan) in Lamp1 positive phagolysosomes (purple), stained as for Fig. 3. Co-localization of PPs with conidia in phagolysosomes is marked with white arrows

The percentage of phagolysosomes containing both PPs and conidia was calculated from CLSM images. For this purpose, different concentrations of PPs were used. As shown in Fig. 5A, a higher concentration of PPs correlated with an increasing number of co-localization events (*p* = 0.0173, *R*^2^ = 0.9993). After 4 h of incubation of macrophages and conidia with 10 μg mL^−1^, 50 μg mL^−1^, and 100 μg mL^−1^ of PPs, we found co-localization of PPs with conidia for 2.8%, 7.6%, and 14.3% of phagolysosomes, respectively. These concentrations of PPs did not affect cell viability after 24 h of incubation (Fig. 5B), nor did they affect the pro-inflammatory cytokine response of infected and uninfected macrophages (Fig. 5C). Other particle sizes were tested in order to exclude that the size plays a role. PPs with a size of ca. 436 nm and 1200 nm were tested for their co-localization with conidia, which was found to be lower than the percentage obtained for particles with a size of ca. 800 nm (Fig. 5D).

Additionally, we tested whether the frequency of the co-localization events can be enhanced with compounds that are known to alter the phagolysosomal trafficking and maturation. As shown in Fig. 5E, only cells treated with vacuolin1 showed significantly increased fusion as compared to the control treated with DMSO. The macrophages treated with vacuolin1 had phagolysosomes of larger volume, as it can be observed in Fig. 5F compared to the control. Also, as expected, we observed reduced fusion events when using wortmannin (Fig. 5E).

## Discussion

The major aim of this study was targeting intracellularly persisting pathogenic microorganisms with PPs. Here, we now provide evidence that targeting phagolysosomes in macrophages already containing microorganisms with PLGA particles is possible. This proof-of-concept opens up several possibilities, e.g., to load such PPs with antimicrobial compounds. Our data strongly suggest that targeting is achieved by the fusion of phagolysosomes containing a conidium with phagolysosomes containing PPs.

A prerequisite for our targeting approach was the generation of fluorescent PLGA particles with a size of > 500 nm to trigger the phagocytosis of PPs (Champion et al. 2008). The formulation technique chosen strongly depends on the properties of the PP material, such as the solubility of the polymer, the requirements of the intended application of the particles, and the desired particle size (Crucho and Barros 2017). The size was controlled by employing a suitable formulation technique and by optimizing parameters: initial concentration, employed solvent, and formulation conditions (Rao and Geckeler 2011). PLGA was the material chosen for this study, as it is one of the most widely investigated and FDA-approved polymer for drug delivery applications (Zhong et al. 2018; Alhakamy and Md 2019). However, with the nanoprecipitation formulation technique, PLGA is known to reach a size limit of less than 500 nm (Huang and Zhang 2018); thus, a single emulsion technique was adopted for the formulation. Particle formation via emulsification entailed two steps: (i) the formation of the organic phase consisting of a polymer and a water-immiscible solvent and (ii) the addition of a stabilizer to the organic phase as well as the application of an external mechanical force by a high-speed homogenizer (Rosca et al. 2004). This method allowed us to obtain a size range of PPs, allowing for their phagocytosis (Lee et al. 2015). The fluorescent labeling of the polymer with DY-550 was useful to track the uptake and intracellular fate of PPs in macrophages.

On average, each macrophage phagocytosed at least 1 PP within 6 h while reaching the maximum percentage of particle-containing macrophages at 4 h. The uptake of our PPs was lower compared to a previous study that reported the use of particles with a size of 389 nm, but higher compared to microparticles of 6.5 µm (Nicolete et al. 2011). The microparticles were apparently not only hardly internalized but also triggered an undesired immune response (Nicolete et al. 2011). The immunogenicity of macrophages when loaded with PLGA particles in size range from 200 to 1465 nm has been tested by Roberts et al. (2013). They reported neither cytokine nor inflammasome activation. Animal experiments confirmed the safety of these formulations, which allow the recruitment of immune cells without hampering or triggering an exacerbated immune response (Roberts et al. 2013). Also, a study using PLGA microparticles for immunization revealed that particles did not alter M1 macrophages’ primary functions such as the production of pro-inflammatory cytokines, reactive oxygen intermediates, inducible nitric oxide synthase (INOS), cyclooxygenase (COX)-2, and reactive nitrogen intermediates (Lu et al. 2010). The studies aforementioned show that there is no impact of PLGA particles on macrophage function, but degradation products such as lactate are known to suppress pro-inflammatory cytokine response (Errea et al. 2016; Hoque et al. 2014; Chereddy et al. 2018). However, independently of the impact of the PLGA degradation products on macrophages, the immunosuppressed host is nonetheless unable to kill the fungus, and the particle delivery should not be affected, as shown here. Additionally, there are reports demonstrating how lactate triggers β-glucan masking on the cell wall of fungi like *Candida albicans*; this modification of the cell wall allows the fungus to evade the immune response (Ene et al. 2012; Ballou et al. 2017). However, lactate-induced β-glucan masking has not been reported for *A. fumigatus*. Furthermore, the surface of conidia is already largely immunologically inert due to the presence of a layer of melanin and hydrophobic rodlet proteins (Aimanianda et al. 2009).

The PPs produced here were observed in compartments positive for Rab7 and Lamp1, which are known markers of phagolysosomes (Pauwels et al. 2017). This finding indicates that the PPs ended up in mature phagolysosomes. Rab7 is a marker for late endosomes and phagosomes, and it drives the phagosome movement in a centripetal direction to allow for fusion with lysosomes (Harrison et al. 2003). The presence of a Lamp1-positive signal indicates that fusion occurred between a phagosome and lysosomes (Huynh et al. 2007). Because of the size of the generated PPs, their uptake, and the presence of phagolysosomal markers detected in the PP-containing compartment, we can conclude that the uptake of PPs occurs via phagocytosis and that they reside in phagolysosomes.

Here, we also provide evidence that PPs can reach phagolysosomes already containing conidia of the pathogenic fungus *A. fumigatus*. This was observed and quantified by immunofluorescent imaging based on the detection of Lamp1 as a marker for phagolysosomal membranes and was further confirmed by live cell imaging and TEM. It was essential for us to simulate physiological conditions in which conidia were already located inside of macrophages before PPs were added and phagocytosed. Because both PPs and conidia were located in the same phagolysosome, two phagolysosomal compartments must have fused inside the cell. Our results agree well with previous findings showing that *A. fumigatus* conidia hardly impaired the fusion of phagosomes with lysosomes but rather the maturation of phagosomes, thus allowing conidia to survive (Schmidt et al. 2020, 2018; Thywissen et al. 2011). It is important to note that we detected phagolysosomal markers at later time points (after 6 h of infection), while previous studies analyzed endosomal markers within 2 h of infection (Schmidt et al. 2018). In summary, our data indicate the fusion of phagolysosomes as a potential delivery mechanism of PPs to phagolysosomes containing pathogenic microorganisms.

In our attempt to increase the number of fusion events, we found positive results by using higher particle concentrations, 800 nm particle size, and vacuolin1. Increasing particle concentrations increased the number of PPs internalized which in turn led to a higher number of fusion events. Apilimod and vacuolin1 are specific inhibitors of the phosphatidylinositol-3-phosphate 5-kinase (PIKfyve) that phosphorylates phosphatidylinositol-3-phosphate (PI3P) to PI(3,5)P_2_ in endosomes (Cai et al. 2013; Sharma et al. 2019). Calcium channels can assemble at PI(3,5)P_2_ on the cytoplasmic side of the endosomal membrane. These channels can control the volume of the phagolysosome, leading to the fusion and fission of the phagosomal membrane (Cao et al. 2017). When the phagolysosomal pH is low, the mammalian mucolipin TRP channel subfamily (TRPML1) calcium channel is open and calcium-calmodulin downstream signaling leads to the reduction of phagolysosome volume by fission (Cao et al. 2017). Therefore, as a result of inhibiting PIKfyve, we obtained phagolysosomes of larger volume and possibly more PI3P on the membrane. Also, vacuolin1 has been associated with RAB5A activation and homotypic fusion (Lu et al. 2014; Sharma et al. 2019). This could have an effect on early endosomes containing the particles that would fuse with the immature phagolysosome containing the conidium (Schmidt et al. 2018). Other molecules that alter the endosomal trafficking by modifying the intraphagosomal pH are metformin and chloroquine, which are known to increase the pH which potentially triggers fusion with more lysosomes in order to regulate the pH value (Kim and You 2017; Fedele and Proud 2020). Also, a higher pH activates the opening of the P2X4 calcium channel that activates calmodulin to increase fusion (Cao et al. 2017). We also used ivermectin, which is an allosteric activator of the P2X4 channel (Csóka et al. 2018). However, this strategy did not increase fusion in our system. Additionally, IFN-γ is a well-known cytokine that stimulates macrophage activity in many aspects including phagosome-lysosome fusion, but this had no effect either (Kak et al. 2018). In contrast, wortmannin inhibits the phosphoinositide 3-kinase (PI3K), thereby reducing homotypic fusion with early endosomes (Mills et al. 1999). As expected, we observed a reduction in fusion when using wortmannin, but fusion was not completely abolished, which suggests that both homotypic and heterotypic fusion still occur.

Our findings open up the possibility to enhance the fusion of phagolysosomes containing pathogens with PP-containing phagolysosomes. Moreover, understanding the mechanisms of subcellular delivery of PPs via phagocytosis and phagolysosome fusion is of relevance to optimize PPs formulations. This way, the outgrowth of pathogenic microorganisms could be prevented at a very early stage of infection, while at the same time, the delivery of antimicrobial drugs to infected macrophages can be expected to minimize off-target effects. Additionally, encapsulating antifungal drugs in PLGA particles could improve the pharmacokinetics of certain drugs (Zolnik and Burgess 2007), e.g., by increasing the solubility of itraconazole at low pH values. The pharmacological and chemical properties of the drug of choice as well as the intraluminal pH of the target organelle must always be carefully considered before encapsulation. Phagolysosomes containing *A. fumigatus* conidia are less acidic, mainly due to the protective melanin layer of the fungus (Schmidt et al. 2020). Therefore, acidification should not be a limiting factor for antifungal therapy with nanoparticles. Our findings might also have implications for the treatment of other pathogenic microorganisms that survive inside macrophages, such as *M. tuberculosis*, *Legionella pneumophila*, *Leishmania* spp., and *Salmonella* spp. (Chifiriuc et al. 2016).

## Supplementary Information

Below is the link to the electronic supplementary material.

Supplementary file1 (PDF 1.65 MB)

## Data Availability

The datasets generated during and/or analyzed during the current study are available from the corresponding author upon reasonable request.
