# Supplementary material for: Targeting of phagolysosomes containing conidia of the fungus Aspergillus fumigatus with polymeric particles
Source: Appl Microbiol Biotechnol. 2022 Dec 8;107(2-3):819–34. doi: 10.1007/s00253-022-12287-1 (PMC9842589; doi:10.1007/s00253-022-12287-1)
Supplement: Supplementary file 1 — Supplementary file1 (PDF 1.65 MB) [file 253_2022_12287_MOESM1_ESM.pdf]

Supplementary Information

Targeting of phagolysosomes containing conidia of the fungus *Aspergillus fumigatus* with polymeric particles

Katherine González[§,a,b], Gauri Gangapurwala[§,c,d], Julien Alex[§,c,d], Antje Vollrath[c,d], Zoltán Cseresnyés[e], Christine Weber[c,d], Justyna A. Czaplewska[c,d], Stephanie Hoeppener[c,d], Carl-Magnus Svensson[e], Thomas Orasch[a], Thorsten Heinekamp[a], Carlos Guerrero-Sánchez[c,d], Marc Thilo Figge[b,e], Ulrich S. Schubert[c,d], Axel A. Brakhage[a,b] *

[a]*Department of Molecular and Applied Microbiology, Leibniz Institute for Natural Product Research and Infection Biology - Hans Knöll Institute (Leibniz-HKI), Adolf-Reichwein-Straße 23, 07745 Jena, Germany*
[b]*Institute of Microbiology, Friedrich Schiller University Jena, Neugasse 25, 07745 Jena, Germany*
[c]*Laboratory of Organic and Macromolecular Chemistry (IOMC), Friedrich Schiller University Jena, Humboldtstraße 10, 07743 Jena, Germany*
[d]*Jena Center for Soft Matter (JCSM), Friedrich Schiller University Jena, Philosophenweg 7, 07743 Jena, Germany*
[e]*Applied Systems Biology, Leibniz Institute for Natural Product Research and Infection Biology - Hans Knöll Institute (Leibniz-HKI), Adolf-Reichwein-Straße 23, 07745 Jena, Germany.*

§ These authors contributed equally to the study
* corresponding author

katherine.gonzalez@leibniz-hki.de; gauri.gangapurwala@uni-jena.de; julien.alex@uni-jena.de; antje.vollrath@uni-jena.de; zoltan.cseresnyes@leibniz-hki.de; christine.weber@uni-jena.de; justyna.czaplewska@uni-jena.de; s.hoeppener@uni-jena.de; carl-magnus.svensson@leibniz-hki.de; thomas.orasch@leibniz-hki.de; thorsten.heinekamp@leibniz-hki.de; carlos.guerrero.sanchez@uni-jena.de; thilo.figge@leibniz-hki.de; ulrich.schubert@uni-jena.de; axel.brakhage@leibniz-hki.de.

Supplementary materials and methods

Materials

Ethyl acetate was used without further purification. PLGA Resomer® RG 502 H (weight-average molar mass M_w = 7 000 to 17 000 g mol^{-1}, acid terminated) and poly(vinyl alcohol) (PVA, Mowiol 4-88, M_w = 31 000 g mol^{-1}) obtained via partial hydrolysis of poly(vinyl acetate) were purchased from Sigma Aldrich (Taufkirchen, Germany). The dye DY-550 (amino derivative) was obtained from Dyomics (Jena, Germany). *N*-(3-Dimethylaminopropyl)-*N'*-ethylcarbodiimide hydrochloride (EDC×HCl, 98.0%, Alfa Aesar, Haverhill, USA), *N*-hydroxysuccinimide (HOSu, 98%, Sigma Aldrich), triethylamine (TEA, 99.0%, Riedel-de Haën, Seelze, Germany), dichloromethane (CH_2Cl_2, 99.8%, Acros Organics, Fair Lawn, USA) and *N,N*-dimethylformamide (DMF, 99.8%, Acros Organics) were used as received. 8-well µ-slides were purchased from ibidi (Munich, Germany) and Millicell slides from Merck (Darmstadt, Germany). Fluorescein isothiocyanate (FITC) and calcofluor white (CFW) were purchased from Sigma Aldrich. Cell culture supplies, Trypan blue and TrypLE cell-dissociating enzyme were obtained from Gibco. Bovine serum albumin (BSA), saponin, glycine, the secondary antibody goat anti-rabbit (IgG H&L) DyLight 633 conjugate (35563), CellMask™ Deep Red and CellLight™ Lamp1-GFP were obtained from Thermo Fisher Scientific (Dreieich, Germany). The primary antibody rabbit anti-mouse Lamp1 (ab208943) was purchased from Abcam (Cambridge, UK). The primary antibody rabbit anti-mouse Rab7 (D95F2) was obtained from Cell Signaling Technology (Frankfurt, Germany). Vectashield mounting medium was purchased from Biozol (Eching, Germany). Dulbecco's phosphate buffered saline (DPBS) was purchased from Merck (Darmstadt, Germany). Osmium tetroxide, Embed812 resin, 2,4,6-tris(dimethylaminomethyl)phenol (DMP-30), uranyl acetate and Reynold's lead citrate and all embedding media for transmission electron microscopy (TEM) were obtained from Electron Microscopy Science (Hatfield, USA). BEEM® capsules were purchased from Plano (Wetzlar, Germany). Carbon-coated TEM grids were obtained from Quantifoil (Großloebichau, Germany). Enhancers were purchased from: metformin hydrochloride from Fischer Scientific (Bremen, Germany), recombinant mouse interferon-γ (IFN-γ) and apilimod dimesylate from R&D systems (Abingdon, UK) chloroquine diphosphate from Sigma Aldrich (Taufkirchen, Germany), vacuolin1 from Santa Cruz Biotechnology (Heidelberg, Germany), wortmannin and ivermectin from Merck (Darmstadt, Germany).

Synthesis of PLGA-DY-550

The dye-labelled PLGA (PLGA-DY-550) was synthesized according to a carbodiimide method as reported elsewhere (Reul et al 2012). In a two necked flask 1 g (83 µmol) of PLGA, 30 mg (156 µmol) of EDCxHCl and 18 mg (156 µmol) of HOSu were added. Vacuum was applied and the flask was re-filled with argon (thrice). The solids were dissolved in 15 mL of dry CH_2Cl_2 and 64 µL (460 µmol) of triethylamine were added. The solution was stirred for 2 h at room temperature. Separately, 1 mg (1.5 µmol) of DY-550 was dissolved in 1 mL of dry DMF and added into the reaction mixture. The reaction mixture was stirred at room temperature overnight, followed by a dilution with 20 mL of CH_2Cl_2 and was washed with 50 mL of distilled water. The organic phase was dried over Na_2SO_4. The solution was filtered and concentrated under reduced pressure. The residual solution was precipitated in cold methanol (–20 °C) and dried in vacuo to receive the labelled polymer as a red powder. Yield = 66% (658 mg). The ^1H NMR spectrum [ppm] measured with an 300 MHz Bruker Avance I spectrometer, (Bruker BioSpin GmbH, Rheinstetten, Germany) in deuterated dimethyl sulfoxide (DMSO-d_6),: δ = 1.37 – 1.64 (br, 3 H, CH_3), 4.77 – 5.08 (br, 2 H, CH_2), 5.08 – 5. 42 (br, 1 H, CH) ppm. The size-exclusion chromatography (SEC) measurements were performed on an Agilent 1200 series system (Polymer Standards Service GmbH (PSS), Mainz, Germany), equipped with a PSS degasser, a G1310A pump and a Techlab oven set to 40 °C. A G1362A refractive index detector and a DAD G1315D detector were utilized for data acquisition. The used eluent was a solution of 0.21% (w/v) of LiCl in *N,N*-dimethylacetamide (DMAc) at a flow rate of 1 mL min^{-1}. A PSS GRAM guard, a PSS GRAM 30 and a PSS GRAM 1000 column were used in series. The obtained dye labelled PLGA revealed a molar mass of M_n = 20.0 kg mol^{-1} with a dispersity (*Đ*) of 1.94. These values were calculated from a calibration curve prepared with poly(methyl methacrylate) (PMMA) using standards obtained from PSS (Polymer Standards Service GmbH, Mainz, Germany) of narrow dispersity (*Đ*). The fluorescence spectrum of the dye was recorded on a Jasco FP-8300 instrument (Jasco, Pfungstadt, Germany) using a spectroscopy-grade solvent (DMSO) and quartz cuvettes (1 cm pathway). The device was measuring from 540 to 800 nm with a scan speed of 100 nm min^{-1} and a data interval of 1 nm.

Determination of PVA content

To determine the PVA content in the final formulations, 20 µL of NaOH (1 M) were added into 90 µL of the PP suspension and mixed for 15 min using a microplate shaker at 850 rpm at room temperature. Afterwards, 20 µL of a 1 M HCl solution, 60 µL of boric acid solution (0.65 M) and 10 µL of Lugol's solution (0.01 M elemental iodine and 0.016 M of potassium iodide) (Spek et al 2015) were added. This sample was then subjected for absorbance (λ = 650 nm) measurement using the TECAN Infinite M200pro microplate reader (Tecan, Crailsheim, Germany). Regression method from standard calibration curve was then used for final PVA content (c = 0.023 to 3.0% w/v, R^2 = 0.97) (Table S1, Fig. S1, in the SI).

Transfection of cells and live cell imaging

RAW 264.7 macrophages were incubated in 8-well µ-slides and transfected with 50 CellLight Lamp1-GFP viral particles per cell. The cells were incubated overnight to allow adherence at 37 °C and 5% (v/v) CO_2 under humidified atmosphere. *A. fumigatus* conidia were stained with CFW (100 µg mL^{-1}) and added to the transfected cells at an MOI of 5 in DMEM containing 2% (v/v) of FCS. The slide was centrifuged for 5 min at 100 *g* to synchronize the uptake. The cells were incubated to allow for phagocytosis of conidia for 2 h. Then, extracellular conidia were stained with 0.04% (v/v) Trypan blue for 1 min (Liesche et al 2015), the cells were washed twice with PBS, and the PPs were added to a final concentration of 10 µg mL^{-1}. Non-transfected cells were stained with the membrane staining dye CellMask Deep Red according to the manufacturer's recommendations, prior to adding FITC-labelled conidia and PPs to the cells. The uptake of PPs by macrophages containing conidia was monitored by CLSM over 6 h.

Transmission electron microscopy

RAW 264.7 macrophages were cultivated overnight in a 6-well plate at a density of 3×10^6 cells per well. The cells were infected with conidia at a MOI of 5 for 2 h and treated with PPs at a final concentration of 100 µg mL^{-1} for 4 h. After incubation, the cells were rinsed with PBS and detached with 0.5 mL TrypLE per well. The cell suspension was then centrifuged for 2 min at 600 *g* at 4 °C. The supernatant was removed and the cells were re-suspended in 4% (v/v) of glutaraldehyde in PBS for 2 h at 20 °C for fixation. Samples were washed thrice with Dulbecco's phosphate buffered saline (DPBS) for 5 min. After each washing step, the cells were centrifuged for 2 min at 134 *g*. The samples were fixed with a 1% (w/v) osmium tetroxide/DPBS solution for 1 h. After fixation, the samples were washed twice with DPBS for 15 min each, followed by dehydration of the samples using a graded ethanol series with 50%, 70%, 90% and 100% (v/v) ethanol content. Each step involved treatment of the samples for 10 min in the respective ethanol solutions and centrifugation at 134 *g* for 2 min. Thereafter, the samples were infiltrated with Embed812 resin first diluted in a ratio of 2:1 with ethanol for 1 h at RT. The samples were centrifuged for 8 min at 134 *g* and the diluted resin was exchanged by Embed812 mixed with 18 µg mL^{-1} of 2,4,6-tris(dimethyl aminomethyl)phenol (DMP-30) and incubated overnight at RT. Incubated samples were again centrifuged at 134 *g* and incubated further with fresh Embed812 and DMP solutions for another 2 h. The cells were then transferred into BEEM® capsules (Plano) and subjected to a fresh Embed812 and DMP solution and were then cured at 60 °C for 24 h. An RMC PowerTome PT-PC Ultramicrotome (Boeckeler Instruments) diamond knife was used to obtain 100 nm thick slices of the cured, embedded samples. These 100 nm slices were collected on carbon-coated TEM grids (Quantifoil) and were stained with a 4% (w/v) uranyl acetate solution for 20 min and an additional 5 min with Reynold's lead citrate. These were then subjected to transmission electron microscopy (TEM) using a FEI Tecnai G^2 20 equipped with LaB_6 filament operated at an acceleration voltage of 120 kV. Images were acquired on a 1376×1024 pixels MegaView CCD camera (Olympus Soft Imaging Solutions).

Image editing

For immunofluorescence, live cell imaging and TEM, the raw images obtained were cropped and contrast enhanced in order to increase visibility and to concentrate on the important details. This was performed with the ImageJ software (v. 1.53c, National Institutes of Health, Bethesda, USA, https://imagej.nih.gov/ij/).

Evaluation of cytotoxicity

RAW 264.7 macrophages (ATCC TIB-71) were cultivated in flat-bottom 96-well plates at a concentration of 2×10^4 cells per well in DMEM supplemented with 10% (v/v) of fetal calf serum (FCS), 2 mM of L-glutamine and 27.5 µg mL^{-1} of gentamycin at 37 °C with 5% (v/v) CO_2 under humidified atmosphere, and were allowed to adhere to the wells and grow overnight. Next, 100 µL of PP suspensions prepared in DMEM (without phenol red) with 2% (v/v) of FCS in concentrations of 1, 10, 50, and 100 µg mL^{-1} were added to the cells. The cells were incubated for 24 h at 37 °C and 5% (v/v) CO_2 under a humidified atmosphere. Thereafter, resazurin was added to each well to reach a final concentration of 20 µg mL^{-1}. The fluorescence of resorufin, the product of reduction of resazurin using NADH+H^+ (O'Brien et al 2000), was recorded after 4 h of incubation by TECAN Infinite M200pro microplate reader (Tecan, Crailsheim, Germany), excitation with a 560 nm wavelength and measuring emission at 590 nm; these measurements were executed in triplicate.

Cytokine measurement

RAW 264.7 macrophages were cultivated in 24-well plates at a concentration of 3×10^5 cells per well in DMEM supplemented with 10% (v/v) of FCS, 2 mM of L-glutamine and 27.5 µg mL^{-1} of gentamycin at 37 °C with 5% (v/v) CO_2 under humidified atmosphere, and were allowed to adhere to the wells and grow overnight. The macrophages were infected with *A. fumigatus* conidia in medium with 2% (v/v) FCS at an MOI of 5; after a brief centrifugation of 5 min at 100 *g* the cells were incubated for 2 h at 37 °C with 5% (v/v) CO_2 under humidified atmosphere. The particles were added to infected and non-infected cells at a concentration of 100 µg mL^{-1}. The cells were incubated for 6 h more at 37 °C with 5 % (v/v) CO_2. As a control for cytokine release, cells were treated with 500 ng mL^{-1} of LPS. At the end of the incubation, the supernatant was collected, centrifuged with 11,000 *g* for 5 min at 4 °C, to remove particles and cell debris. Then, the supernatant was stored at -80 °C for further analysis. The collected supernatant was tested by enzyme-linked immunosorbent assay (ELISA) to detect the mouse cytokine TNF-α (ELISA MAX™ Deluxe Set, Biolegend).

References

Liesche J, Marek M, Günther-Pomorski T (2015) Cell wall staining with Trypan blue enables quantitative analysis of morphological changes in yeast cells. Front Microbiol 6:1-8. doi: 10.3389/fmicb.2015.00107

O'Brien J, Wilson I, Orton T, Pognan F (2000) Investigation of the Alamar Blue (resazurin) fluorescent dye for the assessment of mammalian cell cytotoxicity. Eur J Biochem 267:5421-5426. doi: 10.1046/j.1432-1327.2000.01606.x

Reul R, Tsapsis N, Hillaireau H, Sancey L, Mura S, Recher M, Nicolas J, Coll J-L, Fattal E (2012) Near infrared labeling of PLGA for in vivo imaging of nanoparticles. Polym Chem 3, 694-702. doi: 10.1039/C2PY00520D

Spek S, Haeuser M, Schaefer MM, Langer K (2015) Characterisation of PEGylated PLGA nanoparticles comparing the nanoparticle bulk to the particle surface using UV/vis spectroscopy, SEC, 1H NMR spectroscopy, and X-ray photoelectron spectroscopy. Appl Surf Sci 347:378-385. doi: 10.1016/j.apsusc.2015.04.071

Supplementary Tables

Table S1. Overview of all particle formulations and their characteristics.

Batch	d_H (nm)	PDI	ζ[b] (mV)	c_{PP} (mg/mL)[a]	Residual PVA% (w/v)
PP 1	697	0.27	–36.7	4.80	n.d.
PP 2	896	0.28	–44.6	1.60	0.2
PP 3	764	0.32	–42.0	1.81	0.6
PP 4*	811	0.22	–22.1	1.26	n.d.
PP 5	794	0.04	–24	3.38	n.d.
PP 6[c]	1200	0.11	–21	9.28	0.78
PP 7[c]	436	0.15	–24	3.98	n.d.
PP 8	920	0.29	n.d.	5.89	n.d.

[a] concentration including PVA
[b] measured in Milli-Q water
[c] prepared with homogenization speed 18,000 rpm for 60 s
* measured with Malvern Zetasizer ultra (change of device)
n.d., not determined

Table S2. Significance testing to determine whether the slope of the fitted logistic function, Eq (1), is different from zero in the dynamics that can be seen in Fig. 2. The *p*-values are calculated using two-sided ANOVA.

	$t \geq 0$ h	$t \geq 1$ h	$t \geq 2$ h	$t \geq 3$ h
μ increasing	$p < 10^{-5}$	$p \approx 0.33$	$p \approx 0.89$	$p \approx 0.275$
σ^2 increasing	$p < 10^{-13}$	$p < 10^{-8}$	$p < 10^{-3}$	$p \approx 0.13$

Supplementary Figures

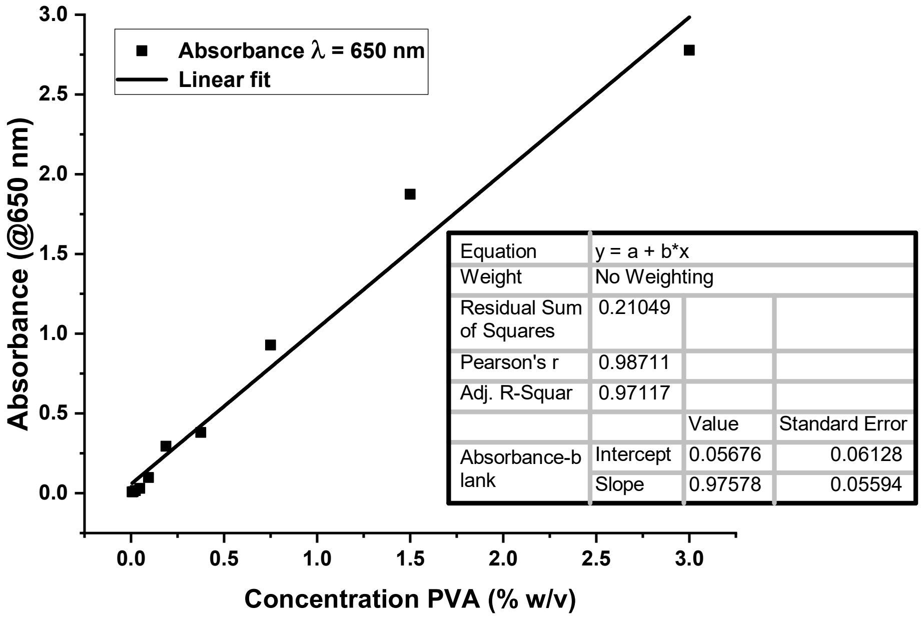

Fig. S1 Calibration curve of PVA dissolved in MilliQ water and after addition of Lugol's solution. The measurement was performed at λ = 650 nm. Method adopted from: Shkodra-Pula B, Grune C, Traeger A, Vollrath A, Schubert S, Fischer D, Schubert US (2019) Effect of surfactant on the size and stability of PLGA nanoparticles encapsulating a protein kinase C inhibitor. Int J Pharm 566, 756-764. doi: 10.1016/j.ijpharm.2019.05.072

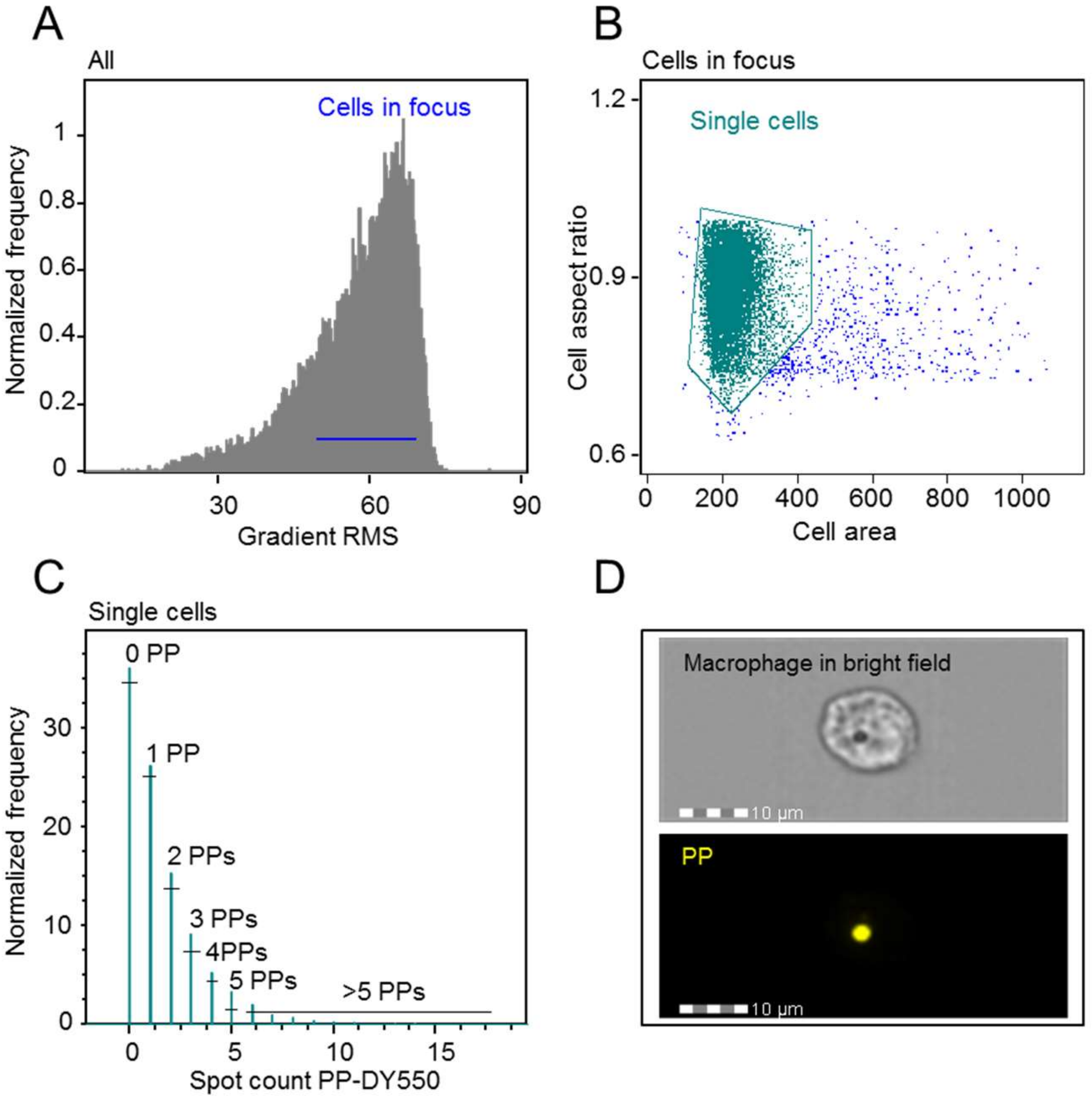

Fig. S2 Gating strategy applied to imaging flow cytometry. **A)** A population of cells in focus were gated based on their high root mean square (RMS) gradient in the bright field channel. **B)** Single cells were gated based on their area and aspect ratio in the bright field channel. **C)** Spot count wizard was used on the channel where the particles had the brightest fluorescence intensity; the graph shows the frequency of the number of particles per cell. **D)** Representative flow cytometry image of a macrophage containing one PP (3 h of incubation)

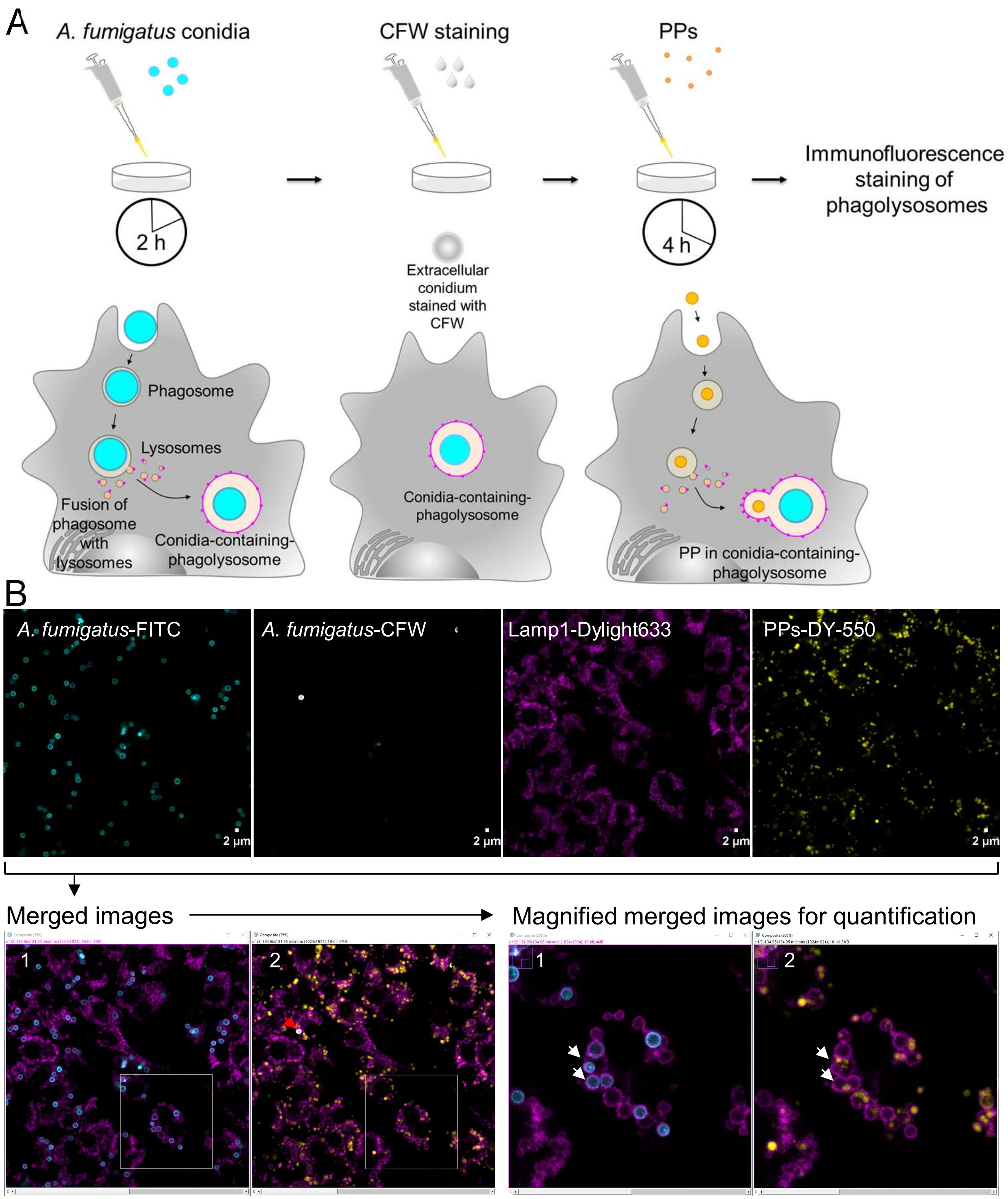

Fig. S3 Strategy for visualization of phagolysosomes containing conidia and/or PPs. **A)** Sequence of infection and staining processes. CFW, calcofluor white; PPs, polymeric particles. **B)** Image analysis in ImageJ. Merge 1: Lamp1-Dylight633 (purple), *A. fumigatus*-FITC (cyan) images merged for visualization of phagolysosomes containing conidia. Merge 2: Lamp1-Dylight633 (purple), *A. fumigatus*-CFW (white) and PPs (yellow) images merged for visualization of phagolysosomes containing PPs. Merged images were compared by visual inspection using the Magnifying glass tool (magnification example in the gray squares). The Multi-point tool was used to quantify the phagolysosomes containing conidia on merge 1 (white arrows, magnified merge) and the phagolysosomes containing PPs on merge 2, that had conidia on merge 1. The phagolysosomes containing conidia stained with CFW (red arrow in merged 2) were not considered for the quantification, since they were outside of the cells when adding the particles

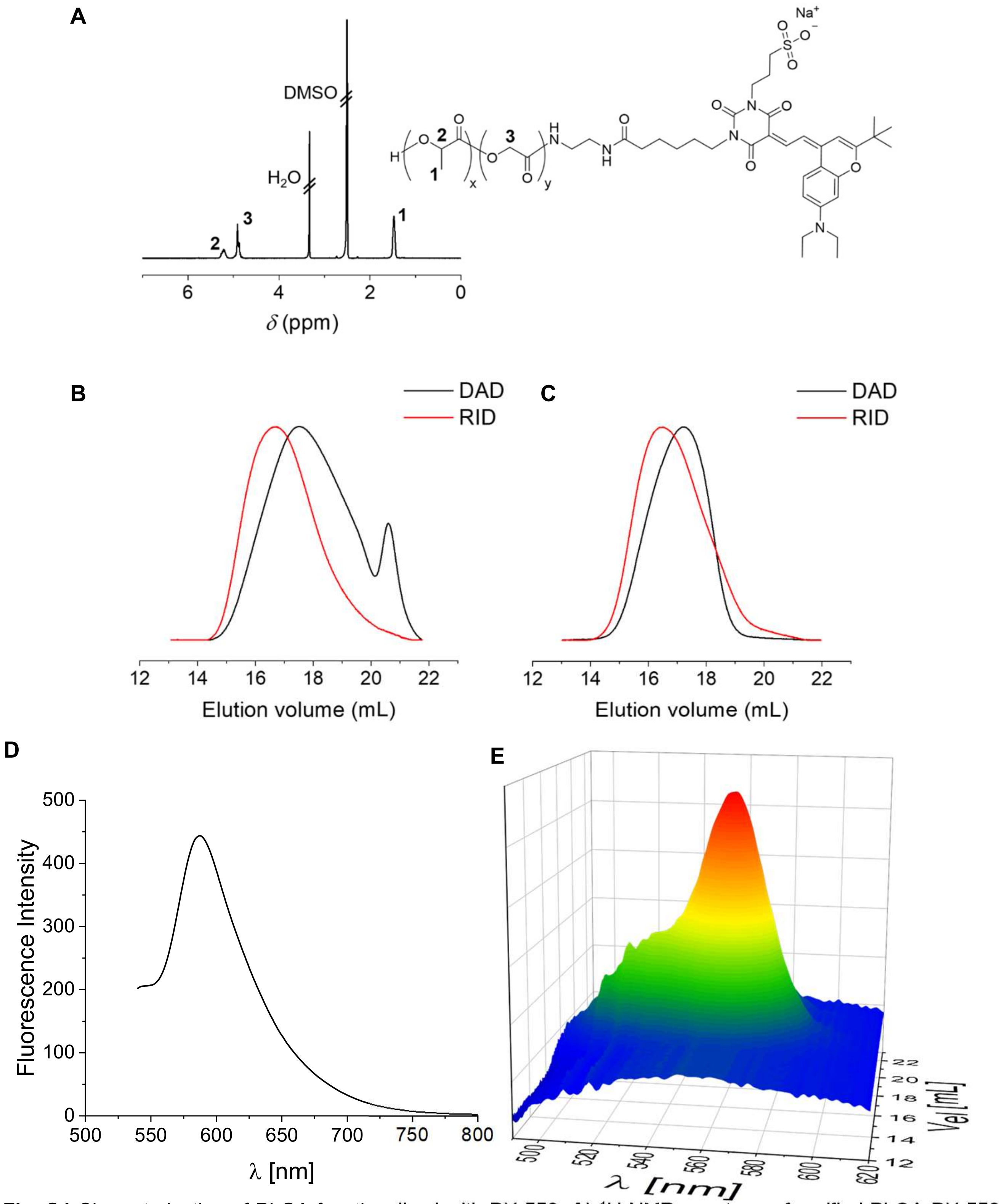

Fig. S4 Characterization of PLGA functionalized with DY-550. **A)** ^{1}H NMR spectrum of purified PLGA-DY-550 (DMSO-d_6, 300 MHz). **B)** Overlay of SEC elugrams of crude reaction mixture recorded by the diode array detector (DAD; absorbance at 550 nm) and refractive index detector (RID), respectively. **C)** Overlay of SEC elugrams of purified PLGA-DY-550 recorded by the diode array detector (DAD; absorbance at 550 nm) and refractive index detector (RID). Spectroscopic characterization of DY-550 functionalized PLGA. **D)** Fluorescence intensity spectrum of the dye-labeled polymer (DMSO; 1.3 mg mL^{-1}, λ_{ex} = 520 nm). **E)** 3D-plot revealing the absorbance of the dye-labeled polymer obtained by SEC measurements (DMAc, DA detection). Both data sets confirm the integrity of the fluorophore after the covalent coupling of DY-550 to PLGA

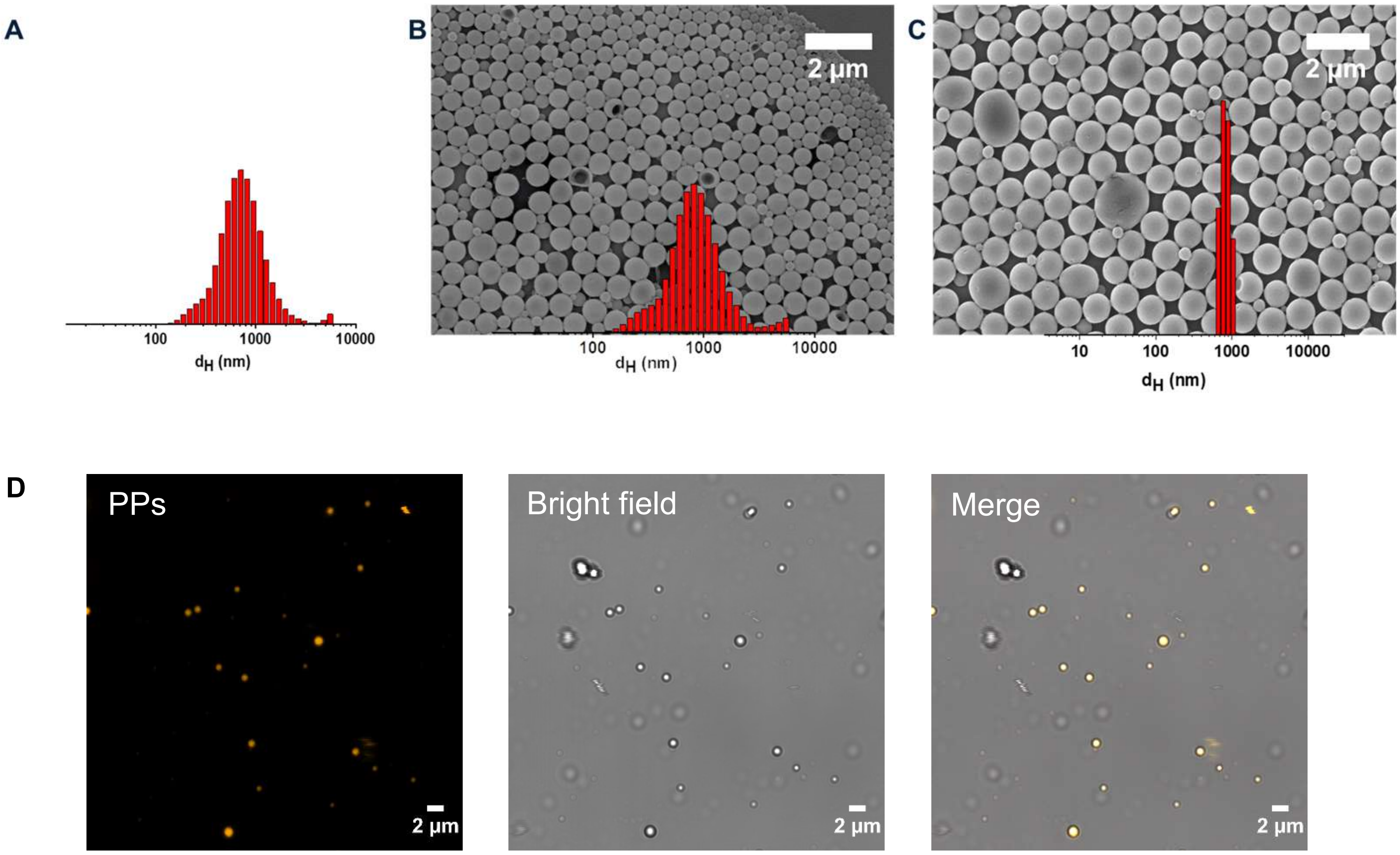

Fig. S5 Intensity size distribution obtained in suspension for **A)** PP 1, **B)** PP 3 and **C)** PP 4 using DLS with cumulant analysis along with SEM image of PP 3 in B) and PP in 4 C). **D)** CLSM images of fluorescently labelled PPs in suspension

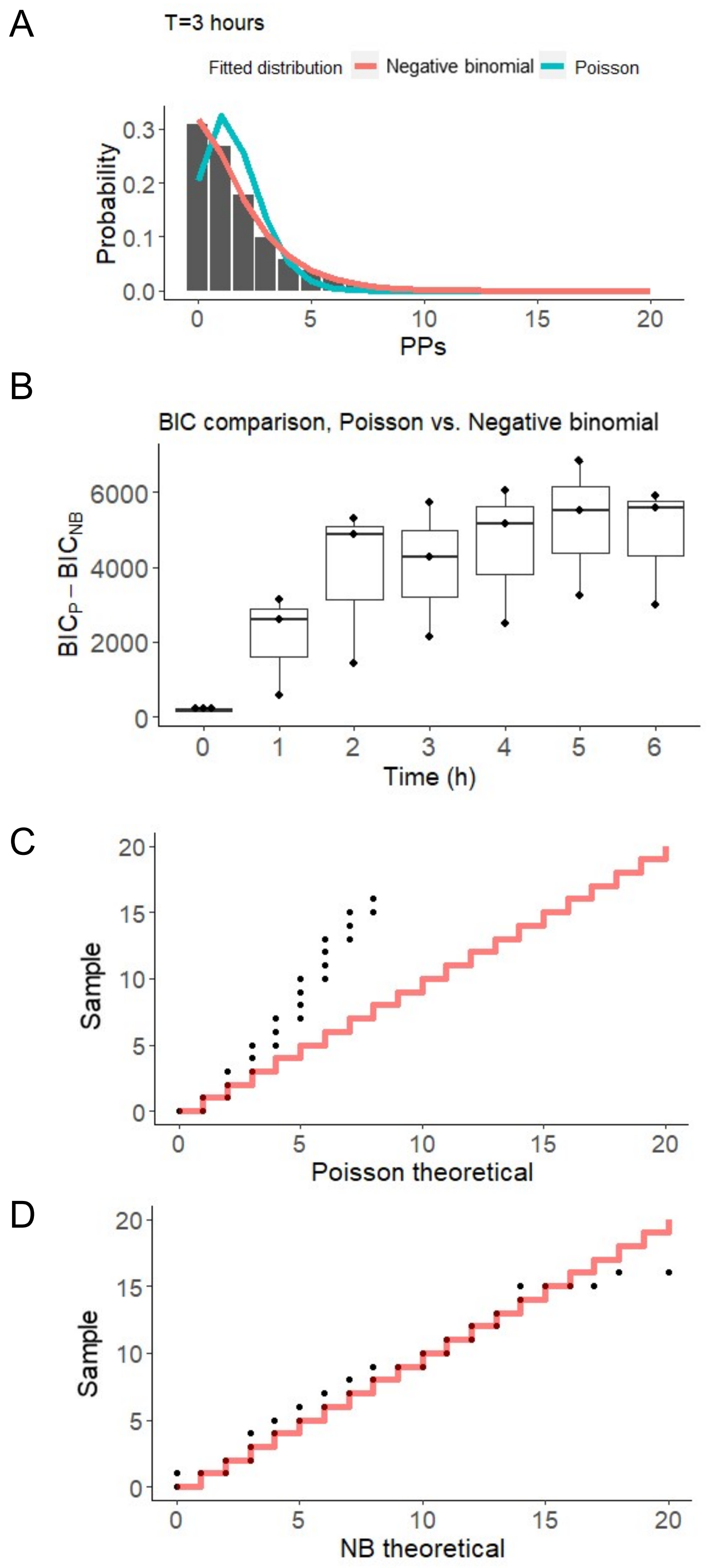

Fig. S6 Dynamics of PP uptake. **A)** Fitting of a Poisson and NB distribution based on the number of PPs per macrophage after 3 hours. **B)** Bayesian information criterion (BIC) difference between the NB fit and Poisson fit. Dots indicate the difference in BIC values for each of the 21 data points. **C)** Q-Q plot of the PP data against a Poisson distribution. Red line indicates the perfect agreement between data and theoretical distribution. **D)** Q-Q plot of the PP data against a NB distribution. Red line indicates the perfect agreement between data and theoretical distribution

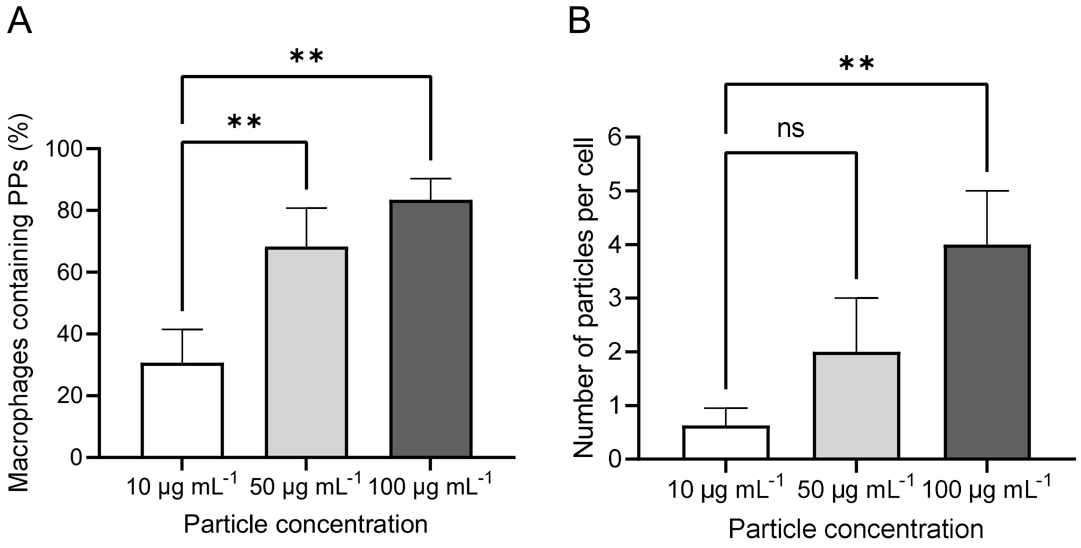

Fig. S7 Particle uptake difference with higher concentration of particles after 2 h of treatment. **A)** Percentage of macrophages with internalized PPs. **B)** Number of particles per cell. Error bars represent standard deviation (n = 3). Replicates were analyzed by one-way ANOVA. Statistical significance: **, $p \leq 0.005$, ns: no significant difference

A B

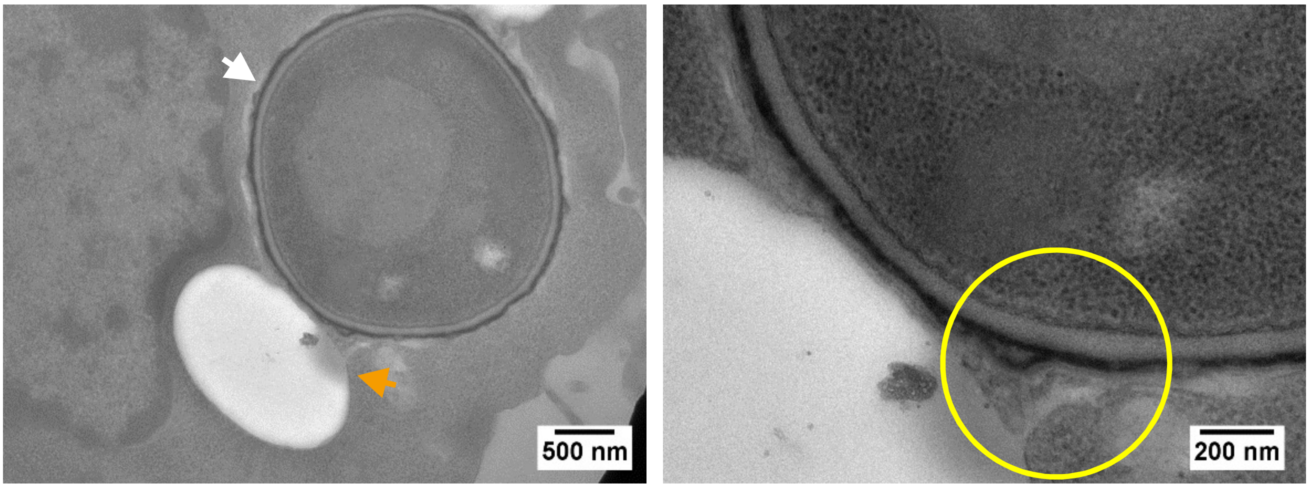

Fig. S8 Transmission electron microscopy image of an apparent membrane fusion. **A)** Conidium-containing phagolysosome (white arrow) fusing with another phagolysosome containing a PP (orange arrow). **B)** The membrane was apparently opened and fusion of both compartments occurred (yellow circle)
